# Predictive value of a fragmented QRS complex in diagnosing patients with myocardial ischemia

**DOI:** 10.1002/clc.23148

**Published:** 2019-02-19

**Authors:** H. J. Cho, J. Y. Yoon, N. Kim, S. Y. Jang, M. H. Bae, J. H. Lee, D. H. Yang, H. S. Park, Y. Cho, S. C. Chae

**Affiliations:** ^1^ Department of Cardiology Daegu Fatima Hospital Daegu Republic of Korea; ^2^ Department of Medicine, School of Medicine Kyungpook National University Daegu Republic of Korea; ^3^ Department of Cardiology, CHA Gumi Medical Center CHA University Gumi Republic of Korea

**Keywords:** fragmented QRS, myocardial ischemia, without scar

## Abstract

**Background:**

A fragmented QRS complex (fQRS) is caused by conduction abnormalities of the ventricle secondary to myocardial ischemia and/or scar in patients with myocardial infarction. However, the implications of the fQRS in the development of coronary artery disease with myocardial ischemia in those without a scar remain unknown.

**Methods:**

We studied electrocardiograms (ECGs) obtained from 150 patients (60.5 ± 8.5 years, 102 men) with myocardial ischemia, which was confirmed by performing both, a nuclear exercise stress test and coronary angiography. We also studied ECGs obtained from 601 patients (58.5 ± 10.0 years, 315 men) who showed a negative nuclear exercise stress test (control group). Patients in whom the nuclear exercise stress test showed a myocardial scar were excluded.

**Results:**

An fQRS was more commonly observed in patients with myocardial ischemia (n = 48, 32.0%) than in the control group (n = 133, 22.1%) (*P* = 0.011). The sensitivity, specificity, positive, and negative predictive values of fQRS in diagnosing myocardial ischemia were 32.0, 77.9, 26.5, and 82.1%, respectively. The fQRS (odds ratio 1.580, 95% confidence interval 1.020‐2.446, *P* = 0.040) was an independent predictor of myocardial ischemia after adjusting for age, sex, current smoking habits, ST‐T changes on ECG, as well as histories of hypertension, diabetes, and dyslipidemia. Moreover, the fQRS showed an incremental prognostic value over conventional risk factors (*χ* ^2^ = 5, *P* = 0.032) and over a combination of conventional factors and ST‐T changes (*χ* ^2^ = 9, *P* = 0.014).

**Conclusions:**

The fQRS is a moderately sensitive and independent predictor of myocardial ischemia.

## 1 INTRODUCTION

Coronary artery disease (CAD) is an important cause of death and morbidity. Early diagnosis and identification of CAD are indispensable, and therefore improved electrocardiographic (ECG) parameters are required for prompt and accurate diagnosis of CAD. A fragmented QRS (fQRS) complex observed in a 12‐lead ECG has been shown to represent inhomogeneous activation of the ventricular myocardium.^1^, ^2^, ^3^ Various prior studies have suggested that myocardial scar tissue is associated with alterations in QRS morphology, leading to a terminal conduction delay, or a fQRS.^4^, ^5^ Previous studies have demonstrated the usefulness of fQRS as a diagnostic tool for the detection of myocardial infarction (MI) and also the prognostic value of fQRS as a predictor of cardiac events including progression of heart failure and death after acute coronary syndrome (ACS). Moreover, fQRS has been shown to be a sign of myocardial scar tissue formation based on myocardial perfusion single‐photon emission computed tomography (SPECT) studies.^6^, ^7^ However, the diagnostic ability of fQRS to predict myocardial ischemia in patients with suspected CAD without myocardial scar tissue remains unknown. Therefore, we investigated whether fQRS can predict myocardial ischemia without scar tissue and whether it shows an incremental value over conventional risk factors including other ECG parameters in patients with suspected CAD.

## 2 METHODS

Among all patients who underwent exercise myocardial perfusion SPECT for evaluation of suspected CAD at Kyungpook National University Hospital (Daegu, Korea) between January 2006 and December 2016, we studied 751 consecutive patients who showed myocardial ischemia on myocardial perfusion SPECT, which was confirmed using coronary angiography (CAG) (ischemia group) and who showed a negative exercise treadmill test and normal myocardium on SPECT (control group). Exclusion criteria were: patients with inconsistent results or myocardial scars observed on an exercise treadmill test and myocardial SPECT, patients with myocardial ischemia on myocardial SPECT, which was not confirmed by CAG (patients did not undergo CAG or no significant coronary artery stenosis was identified using CAG), patients demonstrating a left ventricular ejection fraction (LVEF) <50% observed on echocardiography, and those with a history of myocardial infarction (MI) and/or percutaneous coronary intervention. Demographic and clinical characteristics including age, sex, body mass index, cardiovascular risk factors, and comorbidities (hypertension, diabetes, hyperlipidemia, previous cerebrovascular disease, and current smoking), laboratory tests, LVEF, and ECG findings were recorded. The LVEF was assessed using two‐dimensional echocardiography obtained several days before and after exercise myocardial perfusion SPECT. We obtained 12‐lead ECGs (Philips TraceMasterVue ECG management system, Philips 12‐lead algorithm, Andover, Massachusetts; filter range 0.5‐150 Hz, alternating current filter 60 Hz, 25 mm/s, 10 mm/mV) on the nearest day before and after exercise myocardial perfusion SPECT, and these were analyzed by two independent cardiologists. Disagreements were resolved through consensus. An fQRS was defined as changes in QRS morphology (<120 ms) with diverse RSR′ patterns, that is, additional R (R′) waves, notching, S waves, or > 1 R′ waves in two contiguous leads.^6^ In ECGs showing a bundle branch block pattern with QRS duration >120 ms, fQRS was defined as diverse RSR′ patterns, such as >2 R waves (R′), >2 notches in R waves, or S waves in two contiguous leads.^9^ Those showing incomplete bundle branch blocks were excluded. A pathological Q wave was defined as the presence of a Q wave deeper than one‐fourth size of the voltage of the subsequent R wave, or > 0.04 seconds in duration.^6^ Data are presented as means ± standard deviations for continuous variables and percentages for categorical variables. All comparisons between baseline variables were made using the Student's *t* test for continuous variables and the *χ* ^2^ test for categorical variables. A multivariate logistic regression model was used to identify independent predictors of myocardial ischemia on SPECT. The Cox proportional hazard model was used to evaluate the incremental prognostic values of variables. Incremental variables added to the model at each step were considered significant when the difference in the log‐likelihood values associated with each model corresponded to *P* < 0.05. All *P*‐values were two‐sided, and *P* < 0.05 was considered statistically significant. All statistical analysis was performed using the SPSS software version 20.0 for Windows (SPSS Inc., Chicago, Illinois).

## 3 RESULTS

Of the 751 patients (mean age 58.9 ± 9.7 years, 417 men) who showed myocardial ischemia using myocardial perfusion SPECT, fQRS, and myocardial ischemia were identified in 181 (24.1%) and 150 (20.0%) patients, respectively. Baseline characteristics of patients are shown in Table 1.

**Table 1:** Baseline clinical characteristics of patients with and without myocardial ischemia observed on single‐photon emission computed tomography

	All patients	Myocardial ischemia on SPECT		*P‐*value
	(N = 751)	Yes (n = 150)	No (n = 601)	
Demographics				
Age (years)	58.9 ± 9.7	60.5 ± 8.5	58.5 ± 10.0	0.014
Age ≥ 60 years (%)	373 (49.7)	86 (57.3)	287 (47.8)	0.036
Men (%)	417 (55.5)	102 (68.0)	315 (52.4)	0.001
BMI (kg/m^2^)	24.4 ± 2.9	24.0 ± 2.6	24.5 ± 2.9	0.060
Risk factors and comorbidities				
Hypertension (%)	327 (43.7)	90 (60.0)	237 (39.6)	<0.001
Diabetes (%)	143 (19.1)	43 (28.7)	100 (16.7)	0.001
Dyslipidemia (%)	225 (30.1)	73 (49.0)	152 (25.4)	<0.001
Previous CVD (%)	37 (4.9)	8 (5.3)	29 (4.8)	0.807
Current smoking (%)	159 (21.3)	67 (44.7)	93 (15.4)	<0.001
Laboratory findings				
Hemoglobin (g/dL)	14.0 ± 1.6	14.1 ± 1.7	14.0 ± 1.6	0.679
Creatinine (mg/dL)	0.9 ± 0.9	1.1 ± 1.5	0.9 ± 0.6	0.102
Total cholesterol (mg/dL)	183.7 ± 41.6	180.0 ± 48.4	185.2 ± 38.7	0.288
LDL cholesterol (mg/dL)	118.4 ± 36.8	117.1 ± 40.5	118.9 ± 35.3	0.644
LVEF (%)	60.8 ± 4.8	61.0 ± 4.9	60.7 ± 4.8	0.561
Electrocardiography				
Pathological Q waves (%)	18 (2.4)	5 (3.3)	13 (2.2)	0.378
Atrial fibrillation (%)	12 (1.6)	1 (0.7)	11 (1.8)	0.477
Right bundle branch block (%)	31 (4.1)	3 (2.0)	28 (4.7)	0.143
ST‐T changes (%)	81 (10.8)	23 (15.3)	58 (9.7)	0.045
QRS duration (ms)	91.9 ± 13.6	89.9 ± 10.9	92.3 ± 14.2	0.051
fQRS (%)	181 (24.1)	48 (32.0)	133 (22.1)	0.011

Abbreviations: BMI, body mass index; CVD, cerebrovascular disease; fQRS, fragmented QRS complex; LDL, low‐density lipoprotein; LVEF, left ventricular ejection fraction, SPECT, single‐photon emission computed tomography.

Univariate analysis showed that patients with myocardial ischemia were older and included a greater percentage of men. In addition, this group included a higher percentage of current smokers and those with a history of hypertension, diabetes, and dyslipidemia. There was no difference in body mass index, previous cerebrovascular disease, laboratory findings, and LVEF based on the presence or absence of myocardial ischemia. Among ECG parameters, patients with myocardial ischemia showed a significantly higher percentage of ST‐T changes. However, the pathological Q wave was not statistically significantly different between the groups. Patients with myocardial ischemia identified on SPECT included a higher percentage of current smokers, as well as those with hypertension, diabetes, and hyperlipidemia. The fQRS was more common in the ischemia group (n = 48, 32.0%) than in the control group (n = 133, 22.1%) (*P* = 0.011).

**Figure 1:**
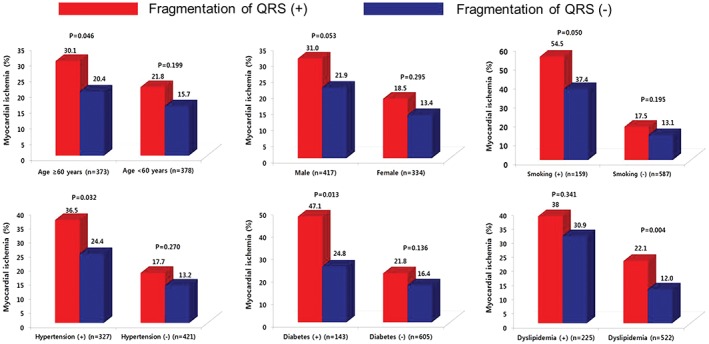
Association between fQRS and the rates of myocardial ischemia based on age, sex, current smoking habits, and histories of hypertension, diabetes, and dyslipidemia. fQRS = fragmented QRS complex

An fQRS was identified on an ECG in 181 patients (24.1%) (Table 2). The fQRS was more common in men and no differences were observed in cardiovascular risk factors, comorbidities, laboratory findings (except hemoglobin), LVEF, and ECG findings in patients with and without fQRS. Myocardial ischemia was more common in patients with fQRS (n = 48, 26.5%) than those without fQRS (n = 102, 17.9%). Subgroup analysis showed that fQRS was a predictor of myocardial ischemia in patients aged ≥60 years, as well as in current smokers and in those with hypertension, diabetes, and a normal lipid profile (Figure 1). An fQRS was more commonly identified in men with myocardial ischemia, although this difference was statistically insignificant.

**Table 2:** Clinical characteristics of patients based on the identification of a fragmented QRS complex

	All patients	fQRS		*P*‐value
	(N = 751)	Yes (n = 181)	No (n = 570)	
Demographics				
Age (years)	58.9 ± 9.7	59.8 ± 9.8	58.6 ± 9.7	0.160
Men (%)	417 (55.5)	116 (64.1)	301 (52.8)	0.008
BMI (kg/m^2^)	24.4 ± 2.9	24.6 ± 3.1	24.3 ± 2.8	0.292
Risk factors and comorbidities				
Hypertension (%)	327 (43.7)	85 (47.0)	242 (42.7)	0.312
Diabetes (%)	143 (19.1)	34 (18.8)	109 (19.2)	0.896
Dyslipidemia (%)	225 (30.1)	50 (27.6)	175 (30.9)	0.400
Previous CVD (%)	37 (4.9)	10 (5.5)	27 (4.8)	0.680
Current smoking (%)	159 (21.3)	44 (24.3)	115 (20.4)	0.258
Laboratory findings				
Hemoglobin (g/dL)	14.0 ± 1.6	14.3 ± 1.5	13.9 ± 1.6	0.024
Creatinine (mg/dL)	0.9 ± 0.9	0.9 ± 0.4	1.0 ± 1.0	0.214
Total cholesterol (mg/dL)	183.7 ± 41.6	185.6 ± 45.7	183.1 ± 40.2	0.574
LDL‐cholesterol (mg/dL)	118.4 ± 36.8	118.4 ± 40.8	118.3 ± 35.3	0.986
LVEF (%)	60.8 ± 4.8	60.4 ± 4.9	60.9 ± 4.8	0.240
Electrocardiography				
Pathological Q waves (%)	18 (2.4)	3 (1.7)	15 (2.6)	0.585
Atrial fibrillation (%)	12 (1.6)	4 (2.2)	8 (1.4)	0.496
Right bundle branch block (%)	31 (4.1)	3 (1.7)	28 (4.9)	0.055
ST‐T changes (%)	81 (10.8)	26 (14.4)	55 (9.6)	0.075
QRS duration (ms)	91.9 ± 13.6	93.1 ± 10.1	91.5 ± 14.5	0.100
Myocardial ischemia on SPECT (%)	150 (20.0)	48 (26.5)	102 (17.9)	0.011

BMI, body mass index; CVD, cerebrovascular disease; fQRS, fragmented QRS complex; LDL, low‐density lipoprotein; LVEF, Left ventricular ejection fraction; SPECT, single‐photon emission computed tomography.

**Figure 2:**
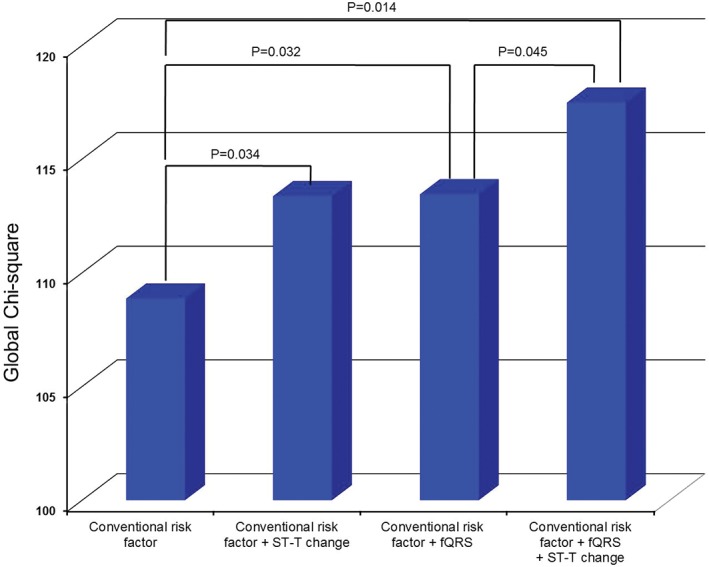
A Cox proportional hazard model shows the incremental prognostic value of ST‐T changes and fQRS. Conventional factors included age ≥ 70 years, heart rate, Killip class ≥2, hyperlipidemia, previous ischemic heart disease, PCI at the time of index hospitalization, left ventricular ejection fraction, and creatinine and hemoglobin levels at the time of admission, as well as aspirin, beta‐blocker, and diuretic use at discharge. fQRS, fragmented QRS complex; PCI, percutaneous coronary intervention

Multivariate logistic regression analysis showed that fQRS (odds ratio [OR] 1.580, 95% confidence interval [CI] 1.020‐2.446, *P* = 0.040) was an independent predictor of myocardial ischemia observed on SPECT after adjusting for age, sex, current smoking, ST‐T changes on ECG, and histories of hypertension, diabetes, and hyperlipidemia. In addition to current smoking (OR 4.666, 95% CI 2.891‐7.532, *P* < 0.001), hypertension (OR 2.050, 95% CI 1.363‐3.083, *P* = 0.001), diabetes (OR 1.600, 95% CI 1.010‐2.535, *P* = 0.045), hyperlipidemia (OR 2.610, 95% CI 1.739‐3.918, *P* < 0.001) and ST‐T changes on ECG (OR 1.840, 95% CI 1.028‐3.293, *P* = 0.040) (Table 3). Moreover, fQRS showed an incremental prognostic value over conventional risk factors (*χ* ^2^ = 5, *P* = 0.032) and over a combination of conventional risk factors and ST‐T changes (*χ* ^2^ = 9, *P* = 0.014) (Figure 2).

**Table 3:** Multivariate logistic regression analysis of factors predicting myocardial ischemia using single‐photon emission computed tomography

	Odds ratio	Confidence interval	*P*‐value
Age	1.025	1.003‐1.048	0.028
Men	1.252	0.780‐2.009	0.353
Current smoking habits	4.666	2.891‐7.532	<0.001
Hypertension	2.050	1.363‐3.083	0.001
Diabetes	1.600	1.010‐2.535	0.045
Dyslipidemia	2.610	1.739‐3.918	<0.001
ST‐T change on ECG	1.840	1.028‐3.293	0.040
fQRS on ECG	1.580	1.020‐2.446	0.040

ECG, electrocardiography; fQRS, fragmented QRS complex.

## 4 DISCUSSION

In the present study, fQRS was an independent predictor of myocardial ischemia without scar tissue. The frequency of occurrence of fQRS on an ECG was significantly higher in patients who showed myocardial ischemia on SPECT than in patients belonging to the non‐ischemic group. Moreover, fQRS identified on an ECG showed a significant incremental diagnostic value over conventional risk factors and ST‐T changes in patients with myocardial ischemia. This result supports the use of fQRS as a novel ECG parameter during situations in which the presence of myocardial ischemia.

Although elective CAG is the best modality to determine the severity of CAD, a CAG is associated with a low diagnostic yield.^8^ Only a third of patients without known disease who undergo elective CAG is likely to be diagnosed with obstructive CAD.^8^, ^9^ Therefore, it is necessary to identify the risk factors to expect CAD before CAG is performed. An fQRS observed on ECG is defined as the unexpected change in QRS complex morphology, that is, fragmentation of the QRS complex.^6^ Several studies suggest that fragmentation of QRS occurs secondary to an alteration in normal ventricular depolarization. Autopsies performed in patients with MI and LV aneurysm have confirmed significant myocardial necrosis, with “islands” of viable myocardial tissue interspersed within abundant fibrous tissue.^10^ These islands of chronically ischemic myocardium display slow activation as a result of partially depolarized and depressed action potential upstroke velocities. This feature causes inhomogeneous ventricular activation with consequent alteration in ventricular depolarization patterns (shown by endocardial mapping and computer models), probably representing QRS complex fragmentation that is identified on the surface 12‐lead ECG.^11^, ^12^ To date, the utility of fQRS has been evaluated in various clinical scenarios in association with necrotic or viable cardiac tissue.^13^

Das et al^6^ reported that fQRS identified on a 12‐lead ECG is a marker of a prior MI, defined as regional perfusion abnormalities and that fQRS shows a significantly higher sensitivity and negative predictive value than that observed with the Q wave. In addition, it has been shown that the presence of fQRS on ECG is associated with a higher incidence of significant CAD than the absence of fQRS in patients with chest pain who are considered to show an intermediate pretest likelihood of significant CAD and would be referred for diagnostic CAG based on a positive stress test.^14^ Gungor et al.^15^ reported that the presence of fQRS on an admission ECG was observed to be a predictor of mortality, major adverse cardiac events, deterioration of LV function, and the presence of multivessel disease in patients with ST‐segment elevation myocardial infarction (STEMI) and non‐STEMI. In addition, Das et al^16^ reported that the fQRS is an independent predictor of mortality in patients with ACS. These previous studies as predictors of CAD primarily focused on the detection of myocardial scars. However, our study showed that myocardial ischemia even without scar tissue is associated with the presence of fQRS on ECG. Korkmaz et al^17^ suggest that the presence of fQRS on ECG is associated with myocardial ischemia in patients with intermediate coronary stenosis with a low fractional flow reserve; however, the absence of myocardial scar was not demonstrated by a functional study such as myocardial SPECT. In our view, our study scores over others in that it demonstrates the utility of fQRS as a diagnostic tool in detecting myocardial ischemia that is proven by both, anatomical and functional modalities/imaging.

### 4.1 Study limitations

Limitations of our study: (a) Ours is a single‐center, observational study; thus, the potential effects of unmeasured or unknown factors on study outcomes cannot be excluded. (b) We did not perform an anatomical study using modalities such as computed tomography angiography for the detection of coronary artery stenosis in the control group. However, we enrolled patients who showed negative results using myocardial SPECT with an exercise treadmill test as the control group, and we consider this was enough to exclude myocardial ischemia.

## 5 CONCLUSION

The fQRS is a moderately sensitive independent predictor of myocardial ischemia. Therefore, using this simple ECG parameter may help to identify patients with suspected CAD who need further aggressive evaluation and treatment.

## CONFLICTS OF INTEREST

The authors declare no potential conflict of interests.
